# Temporal coding of echo spectral shape in the bat auditory cortex

**DOI:** 10.1371/journal.pbio.3000831

**Published:** 2020-11-10

**Authors:** Silvio Macias, Kushal Bakshi, Francisco Garcia-Rosales, Julio C. Hechavarria, Michael Smotherman

**Affiliations:** 1 Department of Biology, Texas A&M University, College Station, Texas, United States of America; 2 Institut für Zellbiologie und Neurowissenschaft, Goethe-Universität, Frankfurt/M., Germany; Universidad de Salamanca, SPAIN

## Abstract

Echolocating bats rely upon spectral interference patterns in echoes to reconstruct fine details of a reflecting object’s shape. However, the acoustic modulations required to do this are extremely brief, raising questions about how their auditory cortex encodes and processes such rapid and fine spectrotemporal details. Here, we tested the hypothesis that biosonar target shape representation in the primary auditory cortex (A1) is more reliably encoded by changes in spike timing (latency) than spike rates and that latency is sufficiently precise to support a synchronization-based ensemble representation of this critical auditory object feature space. To test this, we measured how the spatiotemporal activation patterns of A1 changed when naturalistic spectral notches were inserted into echo mimic stimuli. Neurons tuned to notch frequencies were predicted to exhibit longer latencies and lower mean firing rates due to lower signal amplitudes at their preferred frequencies, and both were found to occur. Comparative analyses confirmed that significantly more information was recoverable from changes in spike times relative to concurrent changes in spike rates. With this data, we reconstructed spatiotemporal activation maps of A1 and estimated the level of emerging neuronal spike synchrony between cortical neurons tuned to different frequencies. The results support existing computational models, indicating that spectral interference patterns may be efficiently encoded by a cascading tonotopic sequence of neural synchronization patterns within an ensemble of network activity that relates to the physical features of the reflecting object surface.

## Abstract 2

Echolocating bats rely upon spectral interference patterns in echoes to reconstruct fine details of a reflecting object’s shape. This study shows that the latency shifts induced by spectral notch patterns can provide the foundation for an avalanche of neuronal synchrony that is sufficient to support encoding of auditory object shape features during active biosonar.

## Introduction

Rate-coding and time-coding strategies are both present in mammalian auditory cortex [1], but the relative costs and benefits of either are still poorly understood, and it is reasonable to predict that the mechanisms and contributions of both are likely to differ across animals and behavioral context [2]. High-precision temporal coding in the mammalian auditory brainstem is essential for sound source localization [3, 4], but there is also general agreement that much of this precision is lost by the time the signal reaches the primary auditory cortex (A1), which instead for most mammals appears to rely primarily upon an unsynchronized rate-coding strategy to encode whole sounds and sequences [1, 2]. An integrative rate-coding mechanism in A1 may be sufficient for processing typical animal communication sounds and even human speech but might not work well for extremely short sounds used for biosonar signals by echolocating bats and cetaceans, many of which are less than a millisecond long. Most bats emit pulses predominated by a very short, broadband, downward frequency-modulated sweep (dFM) when performing echolocation [5, 6]. The main advantage of using a dFM pulse is that this type of signal can deliver an array of critical information in the form of amplitude and spectral modulations, which bats use for detecting, ranging, and classifying targets on a millisecond time scale [7]. Since bats are able to interpret the rapid and precise acoustic cues hidden within brief and faint echoes, they are an ideal model for investigating neural networks underlying temporal coding strategies in mammalian auditory cortex.

Many prior studies investigated how the bat auditory cortex is specialized for detecting echolocation pulses, but few have attempted to address how finely detailed echo acoustic features inside pulses are represented at the cortical level to support complex auditory object recognition. One study identified cortical neurons that responded with a higher spike rate to naturalistic echoes containing spectral interference patterns [8], but stopped short of supporting a rate-coding strategy. The observed rate changes triggered by the appearance of spectral notches appeared to be of insufficient dynamic range or accuracy to support the bats’ known behavioral-discrimination capacities [9–12]. Computational models of biosonar processing instead promoted a time-coding strategy as probably a prerequisite for explaining the remarkable discrimination performance of bats [13]. This led to the hypothesis that the bat A1 might be adapted to rely more heavily upon spike timing than mean spike rates to encode fine echo acoustic details, but stimulus-driven changes in spike timing have not been systematically investigated in the bat A1. If so, the bat A1 could provide novel insights about how cortical networks can be adapted to better preserve and analyze spike-timing information.

The dFM biosonar pulse emissions of bats can be as short as half a millisecond in some bat species (and even shorter in cetaceans) but commonly vary dynamically from about 1 to 10 milliseconds depending on target range [14]. When a biosonar pulse is reflected off of an irregularly shaped surface, multiple overlapping echoes are produced that convolve to generate a single perceived sound with a complex interference pattern of spectral peaks and notches embedded within the short time course of the dFM. The overall pattern of these notches may be unique to each target feature [10, 15, 16]. The spectral notches are typically narrow in bandwidth (<10 kHz) and may be up to 50 dB lower in amplitude than the rest of the echo. An 80-kHz bandwidth echo lasting less than 2 ms could have many shallow spectral notches at bandwidths that are only present within the signal for tens of microseconds. Since it appears bats are able to reconstruct an internal representation of the target based upon these unique spectral interference patterns [10, 15, 17–19], then their auditory system must be able to faithfully encode these rapid modulations. Neurophysiological studies have found evidence that these spectral interference patterns can be encoded by the spike rate of subsets of combination-sensitive neurons found in the inferior colliculus and the auditory cortex of bats [8, 20, 21]. However, although these studies focused mainly on changes in the spike rate of individual neurons, they did not address the neural network mechanisms by which such tuning properties may arise.

Spike timing, especially first-spike latency (FSL), which can contain more or additional information than spike rate, varies with several acoustic parameters including amplitude [22–24], frequency [22, 25], rise-fall time [22, 26], and the velocity and acceleration of amplitude modulations [22, 27]. FSL has been widely explored as a neural code in sensory systems [28, 29]. Spike rate and FSL can vary independent from each other [30, 31] and encode different parameters of an acoustic stimulus [1, 32–34]. However, when representing the same parameters of an acoustic stimulus, FSL appears to be more useful than spike rate [35, 36]. In echolocating bats, FSL can represent both the monaural and binaural intensity cues induced by the head-related transfer function in the peripheral system as a more biologically plausible alternative [37].

Here, we used the A1 of the Mexican free-tailed bat, *Tadarida brasiliensis*, as a model to investigate how spectral shape modulates the spatiotemporal patterns of neuronal activation along the tonotopic axis of A1 and evaluated whether temporal or rate-coding mechanisms, or a combination of both, provided the information needed to support encoding of biosonar target shapes. The Mexican free-tailed bat uses short, broadband dFM pulses similar to other well-studied FM bats [38]. The A1 of the Mexican free-tailed bat is tonotopically organized following the general pattern found in mammals [39, 40]. Neurons tuned to higher frequencies are positioned rostrally and have shorter response latencies than those tuned to lower frequencies [27, 40–46]. Since neurons tuned to higher frequencies have longer latencies, the presentation of a flat-spectrum dFM should cause a smooth sequential activation of neurons in A1 along the anterior-posterior axis. Our hypothesis was that during the response to a dFM with spectral notches, this sequential activation pattern will be disrupted because of the reduced intensities at spectral notches. Amplitude-latency shifts arising in the ascending auditory system result in A1 neurons with characteristic frequencies (CFs) within the spectral notches to fire later in the chain than normal, consequently creating a unique pattern of spike delays. A subsequent processing stage could be wired to detect the emergence of neuronal synchronization between neurons tuned at the notch frequencies and lower-CF neurons. The spectral notch-specific combination-sensitive neurons identified previously in bat cortex [8] could be accounted for by an intracortical neural network wired to detect specific combinations of synchronized firing patterns across A1. Thus, temporal coding based upon synchronization across discreet neuronal populations could therefore provide the mechanism for capturing spectral shape and thereby underlie reconstruction and perception of biosonar target shape. The overall impact of this study derives from the application of recent advances in computational neuroscience to the understanding of how bat auditory cortex performs exceptional functions. The current literature base falls far short of explaining how auditory cortex performs many basic functions, such as how the central auditory system balances the trade-off between temporal and spectral acuity. Specialized systems such as bat biosonar offer clear opportunities to define how general mechanisms can be adapted to perform specialized computation processes. We believe that by shifting the focus from rate coding to temporal coding, the manuscript provides a novel framework for reenergizing research in this field, with impacts extending toward any system that is specialized for processing complex, dynamically modulated sounds such as speech. The results potentially have both biomedical and engineering implications, which add to its long-term impacts.

## Results

### FSL encodes tone frequency and amplitude

**Fig 1:**
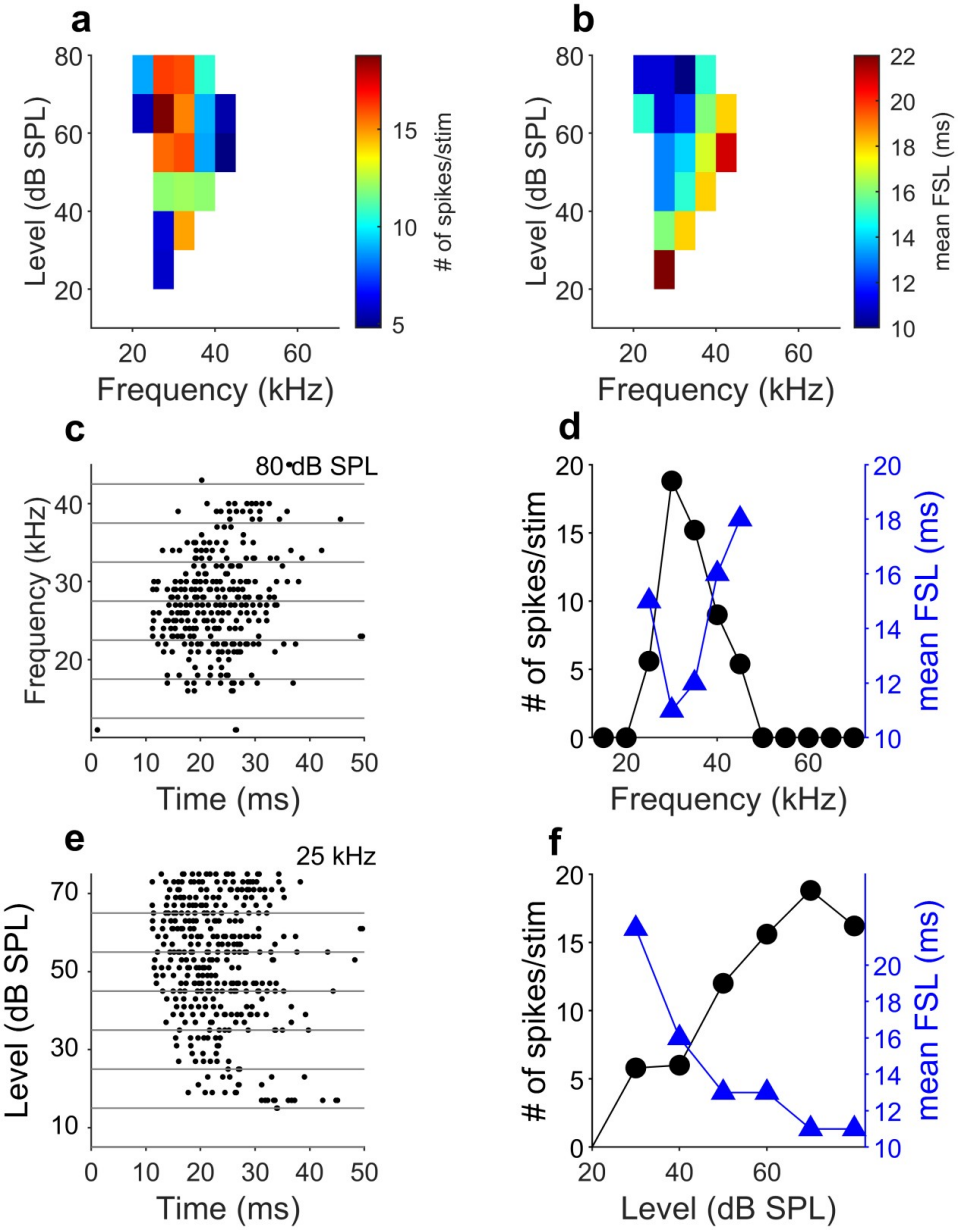
FSL encode sound frequency and amplitude in the primary auditory cortex. (a) Example frequency response area of a cortical neuron (cell: Tb08_1_3) based on the spike number. This neuron is tuned to 25 kHz with a minimum sensitivity of 30 dB SPL. (b) Frequency-latency plot calculated from the mean FSL of the same neurons shown in (a). (c) Dot raster display of the responses at different frequencies at 80 dB SPL. Each dot represents the spike time. (d) Iso-level response function calculated from the spike number (black circles) and the mean FSL (blue triangles) for the responses at 80 dB SPL. The higher number of spikes and the shorter mean FSL occurred at CF. (e) Dot raster display of the responses at different levels at the CF (30 kHz). (f) Spike rate–level function (black circles) and mean FSL-level function (blue triangles). Data underlying this figure can be found in S1 Data.xls at https://doi.org/10.18738/T8/GLVN1J. CF, characteristic frequency; FSL, first-spike latency; SPL, sound pressure level; stim, stimulus.

We estimated frequency representation by both number of spikes and mean FSL by stimulating the bats with 10-ms tone bursts at different frequencies and levels. The frequency response area of an example cortical neuron calculated based on the spike number is shown in Fig 1a. The CF (frequency eliciting the highest number of spikes at the lowest level) of this neuron was 25 kHz with a minimum threshold of 30-dB sound pressure level (SPL). CFs of the neuronal population ranged between 15 and 70 kHz with thresholds between 20 and 60 dB SPL. Mean FSL measured at the CF at 80 dB SPL ranged between 10 and 35 ms. Consistent with previous reports [47, 48], mean FSL was frequency tuned, with the shortest latency occurring at the CF and longest latency at the receptive field periphery (Fig 1b). Fig 1c shows the poststimulus raster figures at different frequencies but the same amplitude (80 dB SPL). Mean FSL was shortest (12 ms) at CF (25 kHz) and lengthened when stimulus frequency differed from CF (Fig 1d). The mean FSL decreased with increasing stimulus amplitude (Fig 1e and 1f).

**Fig 2:**
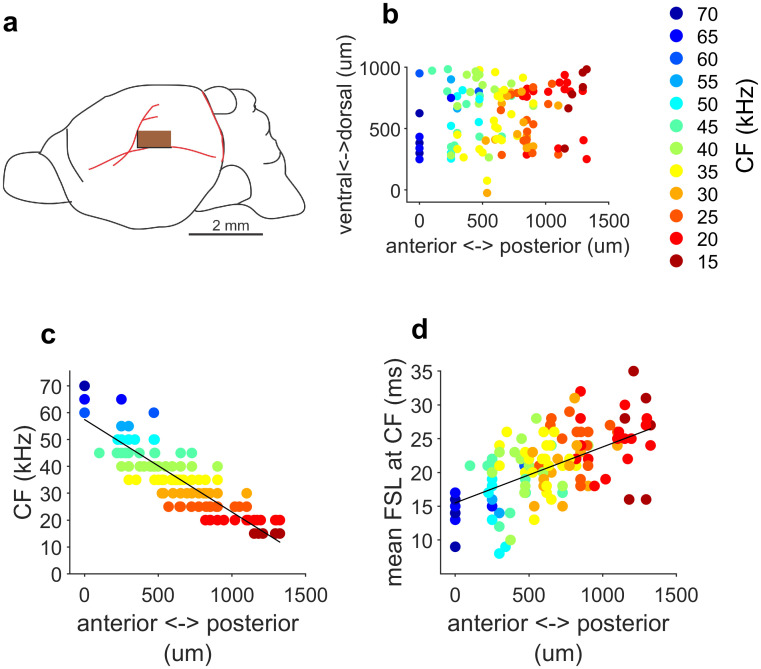
CF and mean FSL are topographically organized in the A1. (a) Schematic drawing of the brain of the Mexican free-tailed bat and localization of the A1. Inset: brown area indicates location of the recorded cortical area. Location of recorded neurons are positioned relative to the major arterial branches (red traces). (b) Tonotopy of the A1. CF are color-coded. (c) CF as a function of the cortical anterior-posterior position. CFs are color-coded similar to (b). (d) Mean FSL calculated at the CF as a function of the cortical location. Colors indicate CF. Black lines represent the best fitted curves of the linear regression analyses. Data underlying this figure can be found in S1 Data.xls at https://doi.org/10.18738/T8/GLVN1J. A1, primary auditory cortex; CF, characteristic frequency; FSL, first latency spike.

Neurons were recorded from a cortical area of approximately 2 mm^2^ (Fig 2a). The frequency representation of the A1 in the free-tailed bat, calculated from data obtained from 122 neurons in the input layer IV (400–600 μm depth) of eight passive-listening bats, is depicted in Fig 2b. Frequency representation of each individual bat is shown in S1 Fig. Neurons tuned to higher frequencies are located in more rostral (anterior) positions with a gradual decrease of the CF in the caudal (posterior) direction (Fig 2c; Pearson correlation, R = −0.87; *p* < 0.05; *n* = 122 neurons). Consequently, as CF decreases along the anterior-posterior axis, mean FSL increases (Fig 2d; Pearson correlation, R = 0.55; *p* < 0.05).

### Amplitude notches in FM sweeps affect neuronal response timing

**Fig 3:**
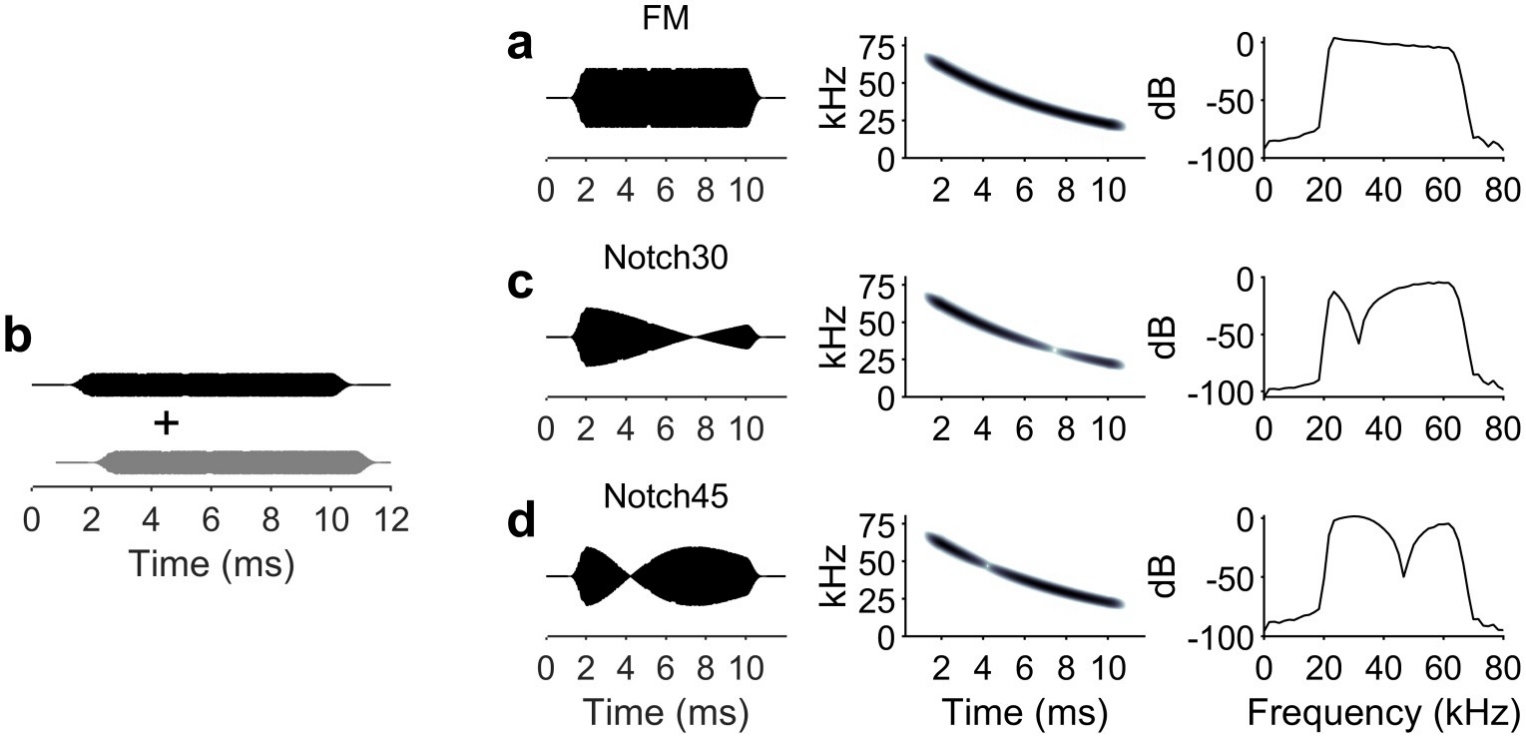
dFMs sweeps used as acoustic stimuli. (a) Flat-spectrum dFM. (b) Sweeps with spectral notches were built by adding two flat-spectrum dFM, where one of them was delays relative to the onset of the other. (c) Spectrally notched dFM resulting from adding two dFM with a 16-μs delay, which turned into a single dFM with a notch at 30 kHz. (d) Spectrally notched dFM resulting from adding two dFM with a 32-μs delay, which turned into a single dFM with a notch at 45 kHz. For (a), (c), and (d) are shown the waveform (left), spectrogram (center), and power spectrum (right). Data underlying this figure can be found in S1 Data.xls at https://doi.org/10.18738/T8/GLVN1J. dFM, downward frequency-modulated sweep.

To investigate the effect of spectral amplitude notches on the response patterns of cortical neurons, we compared the responses to stimulations with a flat-spectrum dFM pulse sweeping from 70 to 20 kHz in 10 ms (Fig 3a) in 157 neurons to those obtained using a stimulus created by convolving two partially overlapping dFM signals, mimicking the summated waveform created by a reflection from an object that contained two surfaces. This simulation was achieved by digitally convolving two dFM signals as described previously (Fig 3b) [8, 20]. We used two different stimuli mimicking echoes representing two different spatiotemporal combinations (16- and 32-μs time delay intervals), which rendered two FM signals with amplitude notches of approximately 30 dB, at 30 kHz and 45 kHz, respectively (Fig 3c and 3d). About 77% of the sampled neurons (122/157) responded to a flat-spectrum or notched dFM signal. The remaining 35 neurons did not respond to either the flat-spectrum dFM or to the notched dFMs.

**Fig 4:**
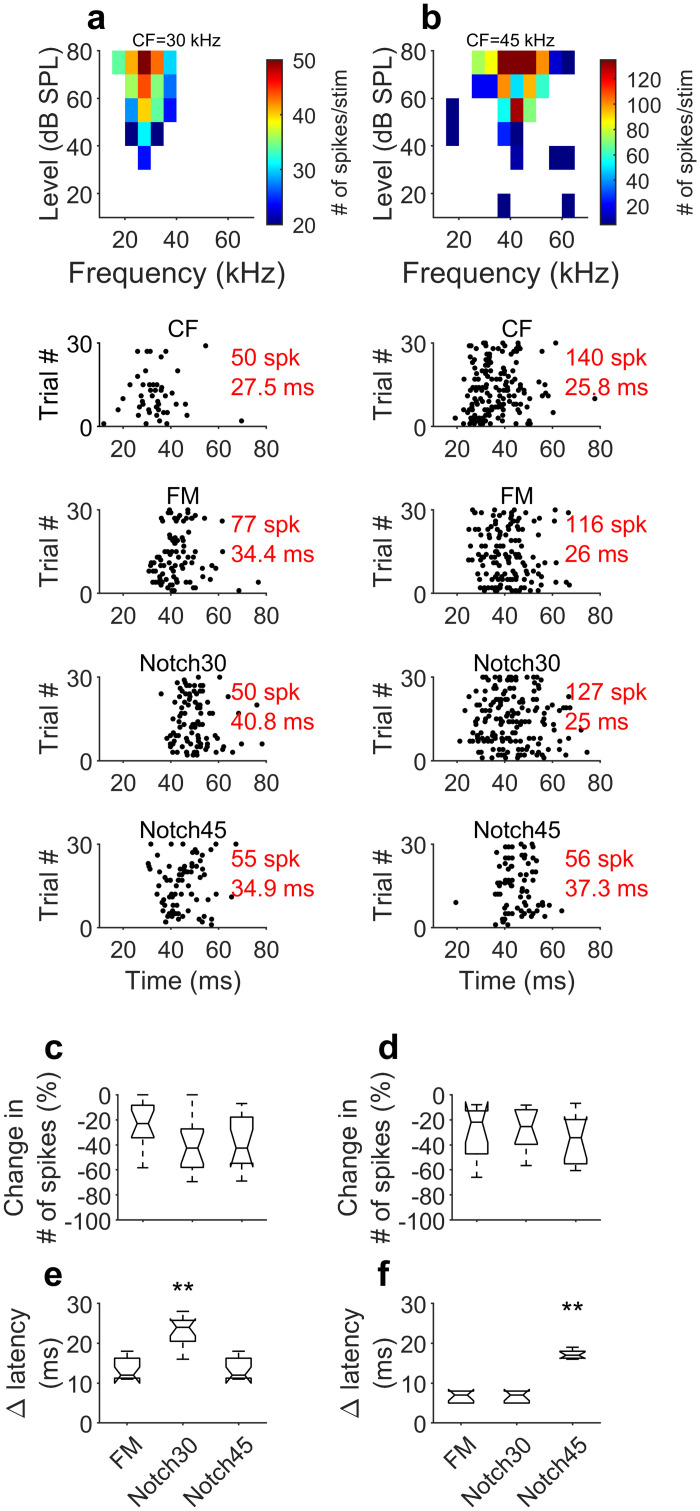
Effect of spectral notches in the response of individual cortical neurons. (a) Example frequency response are of a neuron tuned to 30 kHz (Tb14_2_5) and dot raster displays of this neuron in response to the CF, the flat-spectrum dFM (“FM”), dFM with a notch at 30 kHz (“Notch30”), and a dFM with a notch at 45 kHz (“Notch45”). (B) Example frequency response are of a neuron tuned to 45 kHz (Tb14_18_22) and dot raster displays of this neuron in response to the CF, the flat-spectrum dFM (“FM”), dFM with a notch at 30 kHz (“Notch30”), and a dFM with a notch at 45 kHz (“Notch45”). (c) Comparison of the change in number of spikes in response to the dFMs relative to that elicited in response to the CF in 16 neurons tuned to 30 kHz. Number of spikes at CF is considered 0. Negative percentages indicate lower number of spikes. (d) Comparison of the change in number of spikes in response to the dFMs relative to that elicited in response to the CF in 16 neurons tuned to 45 kHz. Number of spikes at CF is considered 0. (e) Comparison of the change in mean FSL in response to the dFMs relative to that elicited in response to the CF in 16 neurons tuned to 30 kHz. Mean FSL at CF is considered 0. (f) Comparison of the change in mean FSL in response to the dFMs relative to that elicited in response to the CF in 16 neurons tuned to 45 kHz. Mean FSL at CF is considered 0. Box and whisker plots show the median (50th percentile) inside the box delimited by the 25th and 75th percentiles with whiskers extending to the 10th and 90th percentile. Data underlying this figure can be found in S1 Data.xls at https://doi.org/10.18738/T8/GLVN1J. CF, characteristic frequency; dFM, downward frequency-modulated sweep; FSL, first-spike latency; spk, spikes; SPL, sound pressure level.

We compared the responses of the neurons tuned to 30 and 45 kHz (16 and 11 neurons, respectively) to a 10-ms tone burst of their CF to those elicited by each of the three dFMs. Two examples of these neurons are represented in Fig 4. Fig 4a shows the frequency response area of a neuron with a CF of 30 kHz. It also represents the dot raster displays of the responses to the CF, the flat-spectrum (“FM” in figures), the 30-kHz (“Notch30”), and the 45-kHz (“Notch45”) notched dFMs. The mean FSL in response to the dFMs were longer than that measured in the response to the CF, probably reflecting the time delay after stimulus onset when the sweep reached the CF of the neuron. However, the mean FSL calculated in response to the 30 kHz notched dFM was longer than that calculated for the other two dFMs. The neuron tuned to 45 kHz (Fig 4b) showed a lower number of spikes in response to the dFM with a notch at its CF and also a lengthening of the mean FSL of around 10 ms compared to the response to the CF and the two other dFMs.

We compared the change in the number of spikes in response to the dFMs relative to the CF stimulus in the 16 neurons tuned to 30 kHz (Fig 4c) and the 11 neurons tuned to 45 kHz (Fig 4d). In both cases, the dFM with the amplitude notches did not elicit a significantly different number of spikes than the other two dFMs (one-way ANOVA and post hoc Tukey test; *p* < 0.05). The mean FSL was significantly longer when the neuron was stimulated with a dFM with a notch at the neuron’s CF (Fig 4e and 4f, one-way ANOVA and post hoc Tukey test; *p* < 0.05). Fig 4e and 4f show the changes in milliseconds of the mean FSL in the responses to the FM sweeps relative to that measured in the response to the CF. This indicates that the presence of an amplitude notch in the dFM corresponding to the CF of the neuron caused a substantial and significant lengthening of the response latency.

### FSL shift carry more information about spectral notches than spike rate

We computed mutual information (MI, see Materials and methods) between stimuli and responses. MI was calculated when considering spike rate, FSL, and the combination of both spike rate and FSL (joint code). All analyses were based on a poststimulus window of 80 ms. For spike rate coding, the response on each trial included the number of spikes occurring in the time window. For the FSL, the window was subdivided into bins of 2 ms.

**Fig 5:**
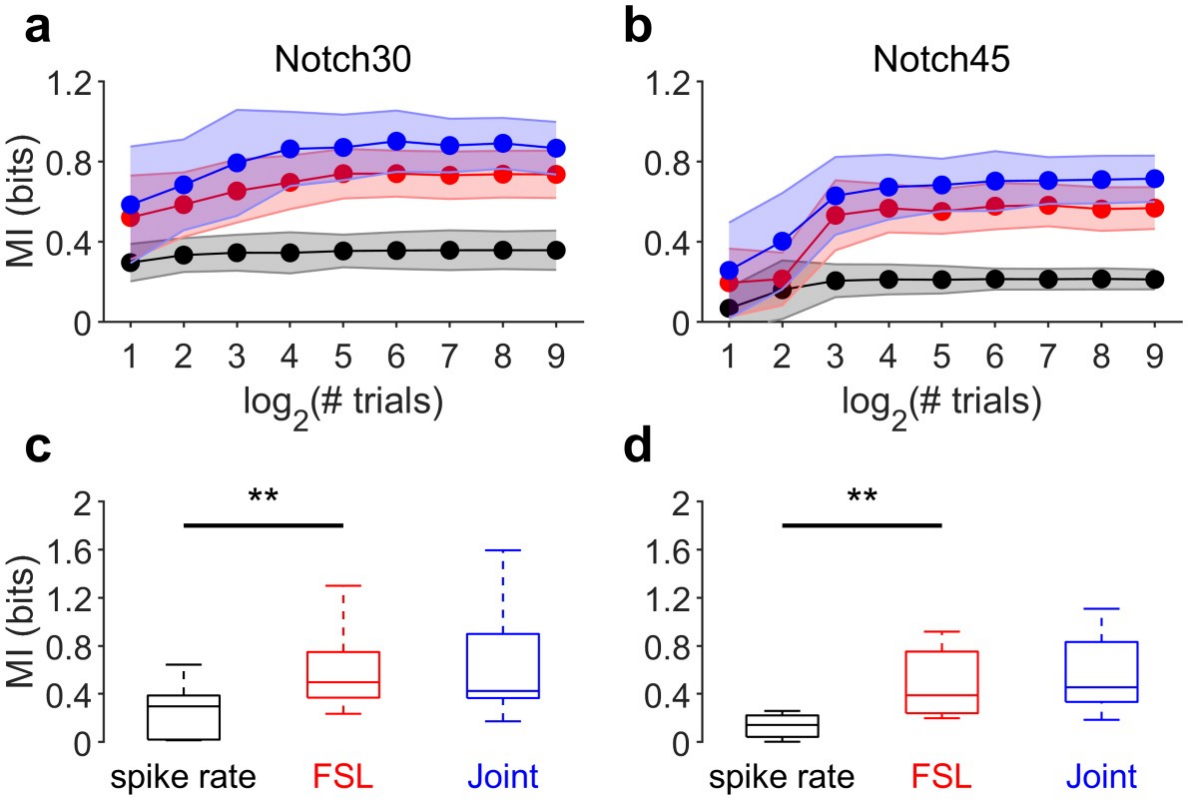
Mean FSL carry more information than spike rate. (a-b) MI and the effect of sample size (# trials) on MI for the Notch30 (a) and Notch45 (b) results. Color scheme is consistent across panels (black represents spike rates, red is FSL, and blue is the combined [joint] value. Performance for bias-correction method used to calculate information values. Data were generated with statistics derived from the real experimental data to assess whether the number of trials included was sufficient for accurate calculation of MI). Calculation of MI was accurate at >15 trials, which was less than the number (30) used in our study. (c-d) Comparison of the MI calculated for spike rates and mean FSL. Notch30 corresponds to the neurons tuned to 30 kHz (*n* = 16). Notch45 corresponds to the neurons tuned to 45 kHz (*n* = 11). Data underlying this figure can be found in S1 Data.xls at https://doi.org/10.18738/T8/GLVN1J. FSL, first-spike latency; MI, mutual information.

The methods used for MI estimation can suffer from bias and the bias depends on the number of trials used: as the number of trials increases, the estimated probabilities become more accurate, and the bias in the MI decreases [49]. The probabilities in calculating the MI must be estimated from a limited number of experimental trials. This can lead to a significant upward bias in the MI. Suppose that a neuron fires in a purely noise way, irrespective of which stimulus occurs (zero MI). This means that the underlying response probabilities will be the same for all the stimuli. Conversely, a neuron that fires selectively to certain stimuli (and does carry information) will have nonuniform probabilities, higher for some stimuli and low for others. The estimation of the set of response probabilities for the noisy neuron from a limited sample of trials will be nonuniform, as if the neuron was genuinely information bearing. As the number of trials increased, the estimated probabilities become more accurate, and the bias in the MI decreases. In order to evaluate the performance of the bias-correction methods used to calculate information values, we generated data with statistics close to the real experimental data and estimated the information in the neural codes following procedures used in previous studies [50] (Fig 5a and 5b). For each neural code (spike rate, FSL, and joint), information was underestimated when fewer than 16 trials were used. However, considering the number of trials used in our recordings (30), the bias is small and does not affect the MI calculation. Fig 5c and 5d show that the MI calculated for the spike rate is significantly lower than those calculated for the FSL and the joint neural codes for both the neurons tuned to 30 kHz and 45 kHz (Kruskal-Wallis one-way ANOVA on ranks and post hoc Tukey test, *p* < 0.05). To sum up, our MI calculations indicate that an external observer could learn more about the dFM stimulus features by looking at the latency information than by considering solely the spike rate and that spike rate and mean FSL together does not provide any additional information.

### Amplitude notches in FM sweeps affect sequential activation of the A1

**Fig 6:**
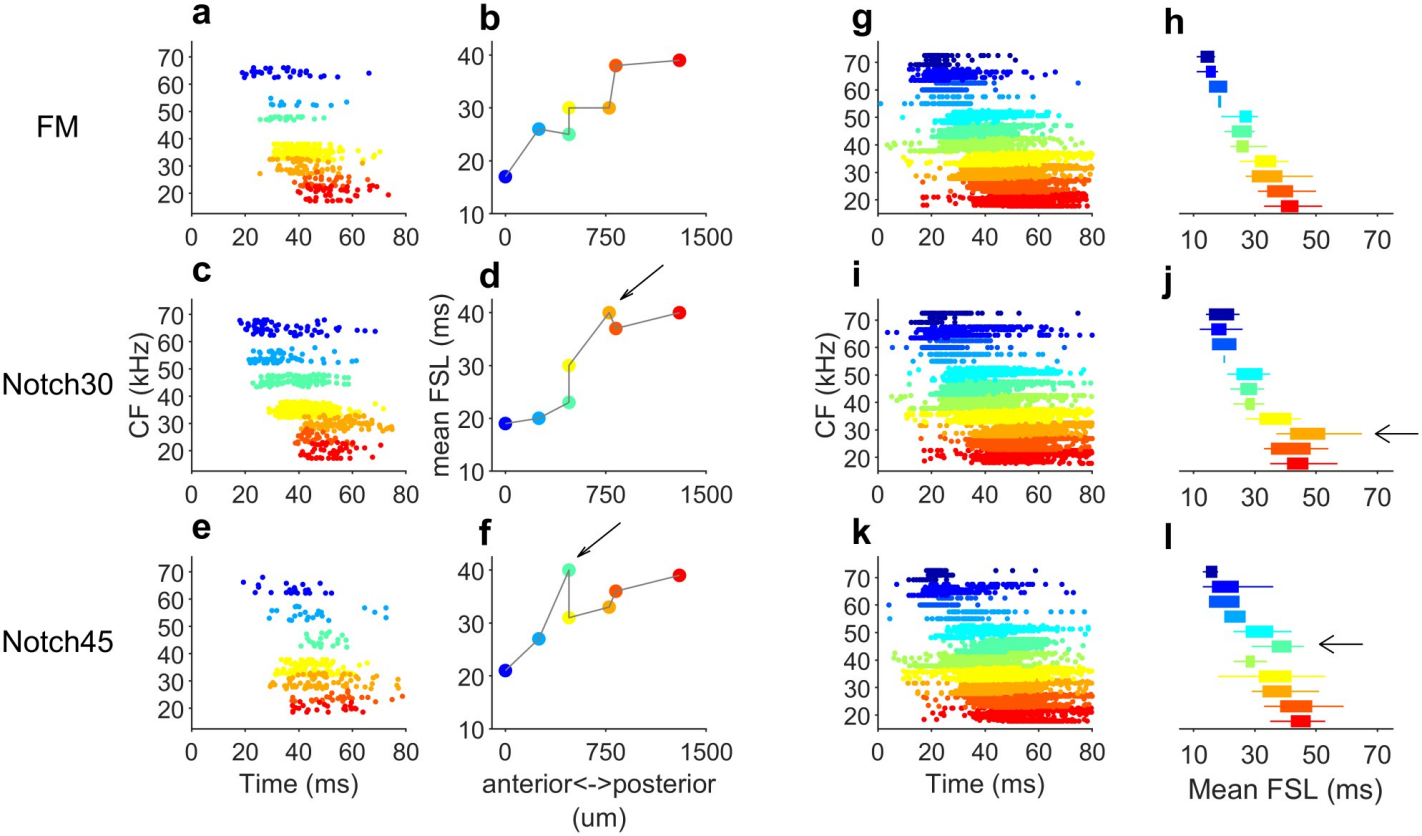
Spectral notches disrupt sequential activation of the A1. (a-f) Tonotopically organized neurons in the A1 of one bat respond sequentially to a flat-spectrum dFMs. (a) Activation pattern profile for this bat shows a sequential activation where anterior-located neurons start responding first. (b) This is illustrated in the mean FSL calculated for each neuron. (c) Stimulation with a dFM with a spectral notch at 30 kHz (“Notch30”) should produce a similar sequential activation; however, the response of the neuron tuned to 30 kHz is delayed. (d) The mean FSL of this neuron is lengthened. (e) A similar activation should happen in response to a dFM with a notch at 45 kHz (“Notch45”). The activation pattern profile is sequential, but the neuron tuned to 45 kHz started responding with a delayed activation. (f) Mean FSL measured in response to the 45-kHz notched dFM. (g) Composite activation pattern profile for all 122 neurons in the five bats in response to a flat-spectrum dFM. (h) Mean FSL calculated from the response of neurons with equal CFs in response to the flat-spectrum dFM. (i) Composite activation pattern profile calculated in response to the 30-kHz notched dFM. (j) Mean FSL for neurons with equal CFs. Arrow indicates the population of neurons tuned to 30 kHz with a lengthened mean FSL. (k) Composite activation pattern profile calculated in response to the 45-kHz notched dFM. (l) Mean FSL for neurons with equal CFs. Arrow indicates the population of neurons tuned to 45 kHz with a lengthened mean FSL. In the activation pattern profiles and the mean FSL, neurons are grouped by color-coded CF following the same conventions as Fig 3. The mean FSL boxplots (h, j and l) represent the median and the 25th and 75th percentile. Data underlying this figure can be found in S1 Data.xls at https://doi.org/10.18738/T8/GLVN1J. A1, primary auditory cortex; CF, characteristic frequency; dFM, downward frequency-modulated sweep; FSL, first-spike latency.

From the above data sets, we constructed neuronal ensemble activation pattern profiles, which took into account the spike times of each recorded cell. Cortical activation pattern profiles were plotted used the individual response of each neuron to each of the acoustic stimuli. Although, in each bat, neurons were not recorded simultaneously, we plotted the spike times of each cell relative to the stimulus onset. These profiles were represented using the response of each cell to the flat-spectrum and notched dFMs. The response of each neuron was organized according to their CF along the tonotopic axis and, therefore, their neuroanatomical position. Neurons at the top of the profiles are tuned to higher frequencies and cells at the bottom are tuned to lower frequencies. In each profile, frequency is color-coded following the conventions in Fig 2. The activation pattern profiles plotted for one bat is shown in Fig 6a–6f. Activation pattern profiles plotted for the remaining seven bats are shown in S2 Fig. In response to a flat-spectrum dFM, the timing of the response of the individual neurons is determined by both the mean FSL and the time the dFM hits the CF of the individual neurons. Consequently, in the activation pattern profile to this dFM, there is a sequential activation of the A1 cortical surface where neurons tuned to higher frequencies, located more anteriorly, are activated first and neurons with lower CF, located more posteriorly, are activated later (Fig 6a). This sequential activation was corroborated by plotting the mean FSL of each neuron versus its anterior-posterior position in the A1 (Fig 6b). In the activation pattern profile in response to a dFM with a notch at 30 kHz, a similar sequential activation of the cortical surface is observable (Fig 6c). However, since there is a lower amplitude at that frequency, a delay in the activation of the neuron tuned to 30 kHz takes place, causing its latency to be lengthened by approximately 10 ms (yellow neuron, Fig 6d). A similar observation is found in response to the 45 kHz notched dFM, as evidenced by the delay in activation of the neuron tuned to 45 kHz (Fig 6e) and lengthening of the mean FSL (Fig 6f).

The disruption of the cortical sequential activation in response to dFMs with amplitude notches was also evident when we pooled the spike times relative to the stimulus onset in all 122 neurons recorded in the five bats. In response to a flat-spectrum dFM, the smooth, sequential activation along the tonotopic axis of the A1 was evident (Fig 6g). This sequential activation was corroborated by plotting the median and the 25th and 75th percentiles of the mean FSL of the neurons tuned to each frequency (Fig 6h). The activation pattern profile built for the responses and the mean FSL calculated to the 30 kHz notched dFM showed an increased latency in neurons tuned to this frequency (Fig 6i and 6j). A similar result was found when plotting the responses to the dFM with a notch at 45 kHz (Fig 6k and 6l).

### Object surface shape is encoded by cortical neuronal synchrony

**Fig 7:**
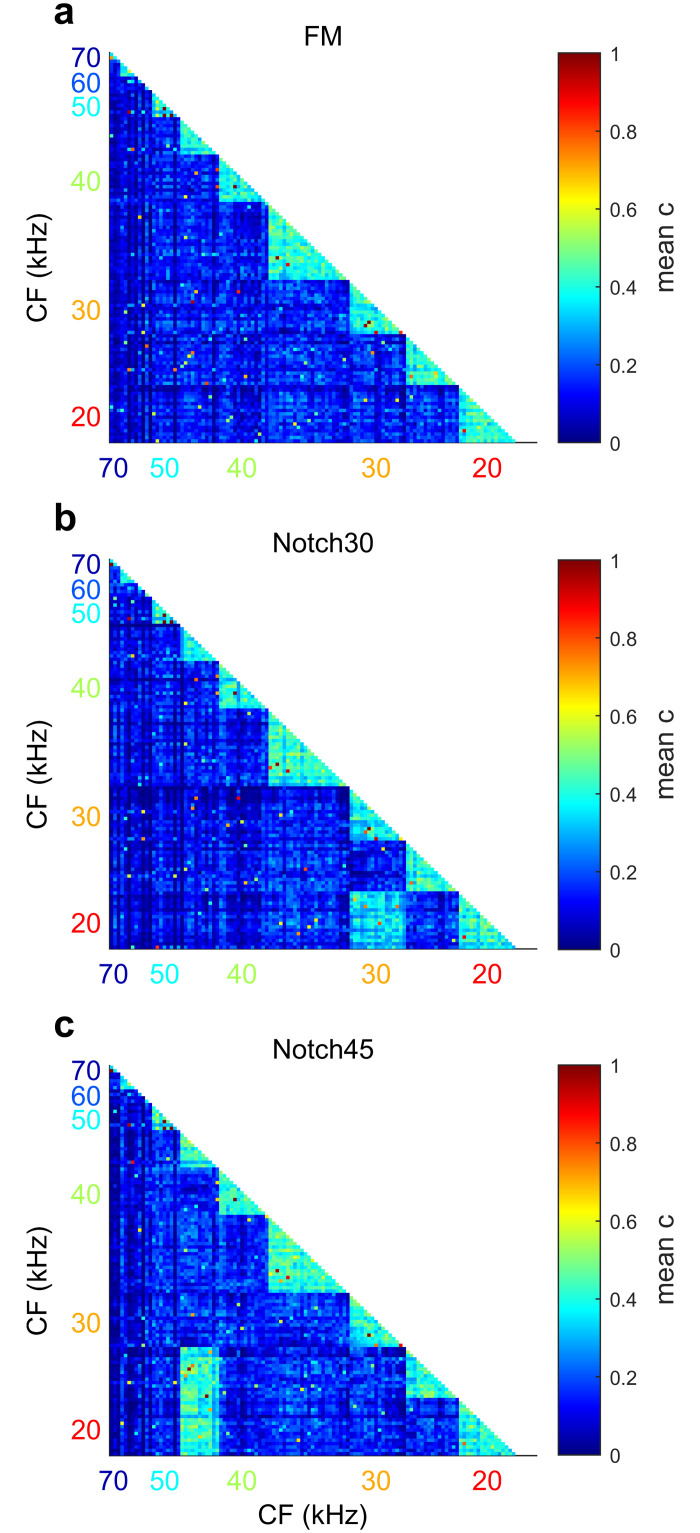
Spectral notches increase neuronal synchronization between neuronal populations. (a) Synchronization index matrix calculated for the response of the neurons in the A1 to the flat-spectrum dFM (“FM”). (b) Synchronization index matrix calculated for the response of the neurons in the A1 to the dFM with a notch at 30 kHz (“Notch30”). (c) Synchronization index matrix calculated for the response of the neurons in the A1 to the dFM with a notch at 45 kHz (“Notch45”). All synchronization matrices were calculated for 122 neurons from eight bats. Synchronization index (c) range from 0 (blue, no spike synchrony) to 1 (red, maximum spike synchrony). Data underlying this figure can be found in S1 Data.xls at https://doi.org/10.18738/T8/GLVN1J. A1, primary auditory cortex; CF, characteristic frequency; dFM, downward frequency-modulated.

Amplitude notches in a dFM delay the activation of the neurons tuned to the notch frequencies, causing their response latencies to coincide with those of cells tuned to lower frequencies and with longer latencies located in more posterior locations in the A1. We hypothesize that this lengthening in the response latencies will increase the neuronal synchronization between neurons tuned to the notch frequencies and lower-CF neurons. From the activation pattern profiles, we quantified neuronal synchronization of all 122 neurons while responding to the dFMs without and with amplitude notches. We evaluated spike train synchrony by using the spike-synchronization index (c), which quantifies the degree of synchrony from the relative number of quasi-simultaneous appearances of spikes [51, 52]. Spike-synchronization is zero if and only if the spike trains do not contain any coincidences and reaches one only if each spike in every spike train has one matching spike in all the other spike trains. To calculate the spike-synchronization index, we used SPIKY [51], a Matlab (MathWorks, Natick, MA) written graphical user interface for monitoring synchrony between artificially simulated or experimentally recorded neuronal spike trains. To test our hypothesis, we calculated synchronization matrices using the spike trains elicited in response to the three dFMs in all the 122 neurons. A matrix was calculated for each trial and from that, we calculated the mean synchronization matrix for each dFM. Synchronization matrices calculated for each bat is shown in S3 Fig. In response to the dFM with no notches, the synchronization index was higher for the spike trains of neurons tuned to the same frequency and lower between neurons with different CFs (Fig 7a). This indicated that neurons with similar CF fire more synchronously than with the other cells. A similar synchronization matrix was obtained for the dFMs with notches. However, an increase in the synchronization index was observed between the spike trains of the neurons tuned to the notch frequency and the spike trains of other groups of neurons tuned to lower frequencies. For example, in the synchronization matrix calculated for the spike trains in response to the dFM with a notch at 30 kHz, there is an increase of the synchronization index between neurons tuned to this frequency and those tuned to 20 kHz (Fig 7b). Similarly, in the matrix calculated in response to the dFM with a notch at 45 kHz, there is an increase in synchronization between the spike trains of the neurons tuned to 45 kHz and those tuned to 20 and 25 kHz (Fig 7c).

## Discussion

It has been demonstrated that encoding information in some cortical sensory structures can include information delivered by the timing of individual spikes [53, 54]. This occurs independently of whether the sensory cortex is probed with a single stimulus characteristic [49] or with a dynamically varying complex stimulus [55–57]. In addition, recent reports have shown that coordinated A1 neuronal activity may be very important for enhanced processing of auditory information [58].

The auditory system of echolocating bats can rapidly extract complex acoustic features within returning echoes that are themselves barely longer than the time course of a single action potential. There is compelling evidence that the bat auditory midbrain relies heavily upon temporal codes, especially FSL [37]. Computational models of the bat auditory system predict that neural circuits arranged to detect synchronized patterns in spike trains across a broad bandwidth can explain how echo features encode cues related to target shape and textures [13], but the location(s) of the networks are still unknown. It is well-established that the bat inferior colliculus uses precise spike timing for duration, interval, and spectral motion tuning [59–61], but there are also indications that the bat auditory cortex may be uniquely wired to integrate spike-timing information [8, 13, 62], although the relative contributions of temporal codes versus rates codes in bat A1 have not previously been evaluated. In this paper, we try to fill that gap by showing that spectral shape information in the bat A1 is better encoded in the spike timing than in the spike rate of individual neurons. In addition, we suggest that complex spectral shapes can be efficiently encoded by the level of synchronized activity present throughout the cortical network.

Neurons in auditory cortex of the big-brown bat showed no or a weak response to flat-spectrum FM signals, and although they displayed an elevated response to FM signals containing naturalistic spectral notches, the dynamic range in firing rates was limited to less than three spikes per echo stimulus [8]. They suggested that this elevated response is related to the existence of both excitatory and inhibitory frequency response areas, a common feature in *Eptesicus* cortical neurons [63]. A flat-spectrum echo will drive both the inhibitory and excitatory frequency regions, resulting in weak or no activity. In contrast, an appropriate notched-spectrum echo (with a spectral notch aligned over the inhibitory response area and energy in the excitatory frequency response area) can disinhibit the neuron, causing it to spike. However, information about the frequency response areas of the sampled neurons and the sensitivity to the partial overlap between the two echoes was lacking. In a survey of the response properties of neurons in the A1 of the Mexican free-tailed bat, the majority of neurons were more sensitive to dFM regardless of whether or not they contained spectral notches [40, 64]. The dynamic range in spiking rates was similarly low in the Mexican free-tailed bat (roughly 0–6 spikes per stimulus) to that reported for the big-brown bat (0–3 spikes per stimulus) even at high amplitudes [8]. This again is inconsistent with any hypothesis that complex spectral shape can be accurately encoded at the cortical level by the spike rates of individual neurons. Furthermore, we found no significant differences in the spike rates elicited by the flat-spectrum dFM and those in response to notched dFMs. However, when the amplitude notch in the dFM coincided with the CF of a particular neuron there was a substantial lengthening of the response latency, even when the spike rate did not differ significantly.

By quantifying the information carried by the spike rate and the mean FSL in response to flat-spectrum and notched dFMs, we found that mean FSL was more informative about the presence of a notch than spike rate. This suggests that mean FSL could play a more significant role in encoding spectral shape at the population level than spike rate does for individual neurons. The significance of spike-timing information varies for different sensory cortices, and at least in somatosensory cortex, timing has been shown to be more informative than spike rate (i.e., stimulus location in the rat barrel cortex) [49]. Preserving the spike-timing information available from even a few spikes, like that produced by the A1, supports the transmission of high quantities of information about behaviorally significant sound characteristics.

We reconstructed the spatiotemporal activation pattern profiles along the tonotopic axis to provide a more comprehensive evaluation of how echo spectral shape was encoded at the ensemble level. The results illustrate that the activation delay caused by a longer response latency led to an increase of synchronization between neurons located at different cortical locations based on their CFs. An essential goal of this network is presumed to be the detection and extraction of complex spectral patterns from within the echo, which is a dimensionality reduction strategy that may be summarized by the activation of combination-sensitive neurons uniquely responsive to particular patterns. Computational modeling [13] predicts that this network is likely to arise from and depend upon circuits wired to detect neuronal synchronization. The first stage of this extraction process might be the synchronization of different types of neuronal subpopulations in auditory cortex. In our study, we used dFM pulses with a single spectral notch each mimicking two dFM separated at 16 and 32 μs, which corresponded to distances of 2.72 and 5.44 mm. It has been extensively documented that object range during echolocation can be encoded by the existence of neurons in the bat midbrain and auditory cortex that are sensitive to call-echo delays [39, 60, 65, 66]. However, these neurons are found to be sensitive to delays between 2 and 20 ms (3.4–340 cm). Here, we suggest that both object surface and very short target distances can be encoded by the increase in synchrony caused by the shifts in mean FSL. We used 10-ms duration dFM. However, during the last stage of acoustic behavior during insect pursuit, bats can shorten the duration of the pulses down to 1 ms [67]. We can speculate, based on our results, that a 1-ms pulse would produce a shorter activation of the A1 and the latency shift would be even more evident.

Synchrony between spatially distributed neurons is required for object representation, response selection, attention, and sensorimotor integration [68–70]. It influences perception, enhancing some representations and suppressing others [71]. There are multiple lines of evidence suggesting that neuronal synchrony plays a role in sensory discrimination and attention. For example, visual discrimination is influenced by synchronized activity in the visual system [71], and eliminating the neural synchrony decreases ability for sensory discrimination [72, 73]. The cortical circuit might use the disrupted activation pattern with higher neuronal synchronization caused by the notch and compare it to a previous activation caused by a flat-spectrum dFM like the previous FM biosonar pulse. The second stage is likely to involve convergence onto higher-order cortical neurons within A1 or in auditory subfields outside of A1. These higher-order cells will respond when specific collections of cells in A1 fire synchronously. These neurons would be predicted to show multipeaked tuning curves and, based on previous studies, would be suspected of being located either on the output layers V or VI of A1 [74–76]. Neurons with multipeaked frequency response areas are common in the auditory cortex [77] and especially in the bat auditory cortex [13, 78], but their spatial patterns and laminar distributions have yet to be characterized. These neurons, wherever they are, become essential to understanding the neural basis for echolocation because they are suspected of playing a central role in the process of encoding spectral interference patterns in echoes [13] and thus capturing clues in echoes about target shape.

## Materials and methods

### Animals

We performed electrophysiological recordings in A1 of eight adult (five females, three males) Mexican free-tailed bats, *T*. *brasiliensis*. Bats were group-housed indoors in an artificial habitat at Texas A&M University (TAMU) with a reversed light cycle.

### Ethics statement

All experiments were carried out according to the National Institutes of Health guidelines and were approved by the TAMU Institutional Animal Care and Use Committee (IACUC Animal Use Protocol #: 2017-0163D).

### Surgical procedures

Animals were anesthetized with a solution of sodium pentobarbital (80 mg/kg) and positioned within a custom-built stereotaxic apparatus. Status of anesthesia was monitored by monitoring breathing and ear-twitch reflexes and maintained at a surgical plane with supplementary doses as needed. Body temperature was maintained within normal ranges using a heating lamp. The skin and temporal muscles overlying the skull were cut and removed, and a custom-fabricated post was attached to the bone at the midline using cyanoacrylate gel. A craniotomy (approximately 2 × 2 mm) was made using a scalpel blade to expose the left auditory cortex.

### Acoustic stimuli

Acoustic stimuli were digitally synthesized and controlled using a custom-written program in Matlab (R2018a, MathWorks, Natick, MA, United States). Sounds were generated at a sampling rate of 250 kHz with a National Instruments card (NI USB-6356, National Instruments, Austin, TX, USA). The audio signal was transferred to an audio amplifier (SONY, STR-DE197, Inwood, NY, USA) and broadcast to the bat with a calibrated ribbon tweeter loudspeaker (Dayton Audio, PTMini-6, Springboro, OH, USA) centered 10 cm directly in front of the head. The calibration curve was obtained with a Brüel and Kjaer sound-recording system (1/4-inch Microphone 4135, Microphone Preamplifier 2670, Brüel and Kjaer, Naerum, Denmark) connected to a conditioning microphone amplifier (Nexus 2690, Brüel and Kjaer, Naerum, Denmark).

To measure the frequency response area, we presented the animal with a pseudorandomized series of pure tones (10-ms duration, 0.5-ms rise/fall time) at different SPLs (step size 10 dB, range 20–80 dB SPL) and frequencies (step size 5 kHz, range 15–70 kHz). Each frequency-level combination was presented five times at an interval of 300 ms. In addition to the pure tones, we presented a downward FM with 50-kHz bandwidth (between 20 and 70 kHz) and 10-ms duration. To test for the effect of spectral notches in dFMs, we added two flat-spectrum dFMs with the second dFM delayed by 16 and 32 μs. This rendered two dFMs with a notch at 35 kHz (Fig 3c) and a second with a notch at 45 kHz (Fig 3d). The three dFMs were presented 30 times at an RMS level of 80 dB SPL and with a time interval of 300 ms.

### Electrophysiological recordings

Experiments were performed in a custom-built sound-attenuating anechoic chamber. Slightly anesthetized bats were placed in a body mold made of soft plastic foam, and the head was tightly affixed to the stereotaxic apparatus by a rod attached to a metal holder. Neuronal recordings were performed using silicon probes from Cambridge Neurotech (16 contacts × 2 shanks per probe with 250 μm between shanks and 50-μm spacing between contact sites along each shank). Each shank had a thickness of 15 μm. Using a micromanipulator system (MX7600R, Siskiyou, Grants Pass, OR, USA), probes were positioned perpendicular to the pial surface based upon landmarks and stereotaxic coordinates and then inserted slowly into the brain through the intact dura mater to a depth of approximately 900 ± 50 μm at the deepest contact point. Neuronal data were acquired with an OmniPlex D Neural Data Acquisition System recording system (Plexon) at a sampling rate of 40 kHz (per channel) and 16-bit precision. Synchronization between the neural recordings and acoustic stimulus broadcasts was achieved with a TTL pulse output from the National Instrument card and recorded on one of the analog channels of the Plexon data acquisition system.

### Analysis of neural recordings

Since we did not find differences in the frequency tuning, bandwidth of frequency response areas, or directional selectivity to the FM sweep across cortical depth [64], in this study we only included data recorded at depths between 400 and 600 μm, corresponding to input layer IV. The raw signal was digitally bandpass-filtered offline (elliptic, second order) between 500 and 3,000 Hz to obtain the multiunit activity. Neural recordings were sorted following methods outlined by [79]. The Wavelet transformation and the superparamagnetic clustering resulted in isolation of single-unit extracellular potentials that matched with qualitative assessments of spike waveforms and estimates of single-unit isolation based on spike refractory periods. Recordings with spike amplitudes lower than four times the amplitude of the recording background noise were not included in the data analysis. From the raster plots, representing the spike time versus the trial number, we measured the number of spikes in a window of 50 ms after the stimulus onset for each frequency-level combination to build the frequency response areas. In each frequency response area, we calculated the CF (frequency eliciting the higher number of spikes at the lowest level). In the responses to the CF at 80 dB SPL and the responses to the dFMs, we measured the number of spikes and the mean FSL. Because some neurons showed some spontaneous firing, we calculated the mean FSL by measuring the time of the first spike after the poststimulus time histogram reached 25% of its peak. This minimized the influence of spontaneous activity spikes on the response times.

Blood vessels around the medial cerebral artery were very consistent from bat to bat. This allowed us to locate the A1 and use the vessels as reference points for stereotaxic measurements. For each bat, coordinates of the recording sites in relation to a branch of the median cerebral artery were measured using a calibrated micromanipulator (MX7600R, Siskiyou). All cortices were aligned together for the construction of composite maps using the branches and the median cerebral artery to determine the orientation of the ordinate axis of the bidimensional Cartesian space of analysis.

In order to quantify how units code spectral shape, we applied information theoretic methods. We calculated MI that quantifies how well an ideal observer of neuronal responses can discriminate between the different stimuli, based on a single response trial [49, 50]. We compared the MI calculated for the spike rate, the mean FSL and the information provided by a joint rate-latency code. The information provided by a joint rate-latency code was calculated taking into account the FSL from individual neurons, on a trial-by-trial basis. Quantified neuronal latencies (binned into 2-ms bins) and firing rate were combined to form two-dimensional response array per unit. Note that the combined response array comprised the same response arrays used to calculate MI considering only rate or latency codes. Joint MI values were bias-corrected by means of the quadratic extrapolation (QE; [80]) and the shuffling procedures [49].

The MI between a stimulus S and response R can be expressed as follows:

I(R;S)=H(R)-H(R|S)

where H(R) is the response entropy (i.e., the total variability of the response distribution) and is calculated as

$$H(R)=-\sum_{r\in R}P(r)log\left[P(r)\right]$$

while

$$H(R|S)=-\underset{s\in S}{\sum }P(s)\underset{r\in R}{\sum }P(r|s)log_{2}[P(r|s)]$$

is known as the “noise entropy” and represents the irreproducibility of the response given a stimulus. The probabilities P(r) and P(s) represent the probability of a particular response in R, and the probability of a particular stimulus in S, respectively, whereas P(r|s) represents the conditional probability of a response r given a stimulus s. To calculate the neuronal responses (spike rate and mean FSL), we considered a time window of 0–80 ms after the stimulus onset. In calculating the information conveyed only by the spike rate, the response r was computed as the number of spikes emitted in this time window on one trial. To study information conveyed by the FSL, we divided the spike trains into bins of 2 ms. Both P(r) and P(s) depend on the assumptions made regarding how the response is quantified and how the stimulus set is defined. Note that the units of MI are bits, given that the logarithm used for the calculations is of base 2. Each bit of information implies that an observer can reduce its uncertainty about the stimulus (based on the response) by a factor of 2. All information analyses were conducted using the Information Breakdown Toolbox (ibTB) [49, 81].

## Supporting information

S1 Fig Topography of CF and mean FSL in the A1. CF and mean FSL as a function of the cortical anterior-posterior position in each bat included in this study. CF is color-coded similar to Fig 2. Black lines represent the best fitted curves of the linear regression analyses. Data underlying this figure can be found at https://doi.org/10.18738/T8/GLVN1J. A1, primary auditory cortex; CF, characteristic frequency; FSL, first-spike latency.
(DOCX)

S2 Fig Sequential activation of the A1. Activation pattern profile and mean FSL in each bat in response to flat-spectrum (left), 30-kHz notched (center), and 45-kHz notched (right) dFM of each bat. Neurons are tonotopically organized. Mean FSL was calculated from the response of neurons with equal CFs. CF is color coded following Fig 2. Data underlying this figure can be found at https://doi.org/10.18738/T8/GLVN1J. A1, primary auditory cortex; CF, characteristic frequency; dFM, downward frequency-modulated sweep; FSL, first-spike latency.
(DOCX)

S3 Fig Synchronization matrices. Matrices were calculated in response to flat-spectrum (left), 30-kHz notched (center), and 45-kHz notched (right) dFM of each bat. Synchronization index (c) range from 0 (blue, no spike synchrony) to 1 (red, maximum spike synchrony). Data underlying this figure can be found at https://doi.org/10.18738/T8/GLVN1J. dFM, downward frequency-modulated sweep.
(DOCX)

## Abbreviations

A1: primary auditory cortex
CF: characteristic frequency
dFM: downward frequency-modulated sweep
FSL: first-spike latency
MI: mutual information
SPL: sound pressure level
