# Supplementary material for: Temporal coding of echo spectral shape in the bat auditory cortex
Source: PLoS Biol. 2020 Nov 10;18(11):e3000831. doi: 10.1371/journal.pbio.3000831 (PMC7678962; doi:10.1371/journal.pbio.3000831)
Supplement: S1 Fig — CF and mean FSL as a function of the cortical anterior-posterior position in each bat included in this study. CF is color-coded similar to Fig 2. Black lines represent the best fitted curves of the linear regression analyses. Data underlying this figure can be found at https://doi.org/10.18738/T8/GLVN1J. A1, primary auditory cortex; CF, characteristic frequency; FSL, first-spike latency. (DOCX) [file pbio.3000831.s001.docx]

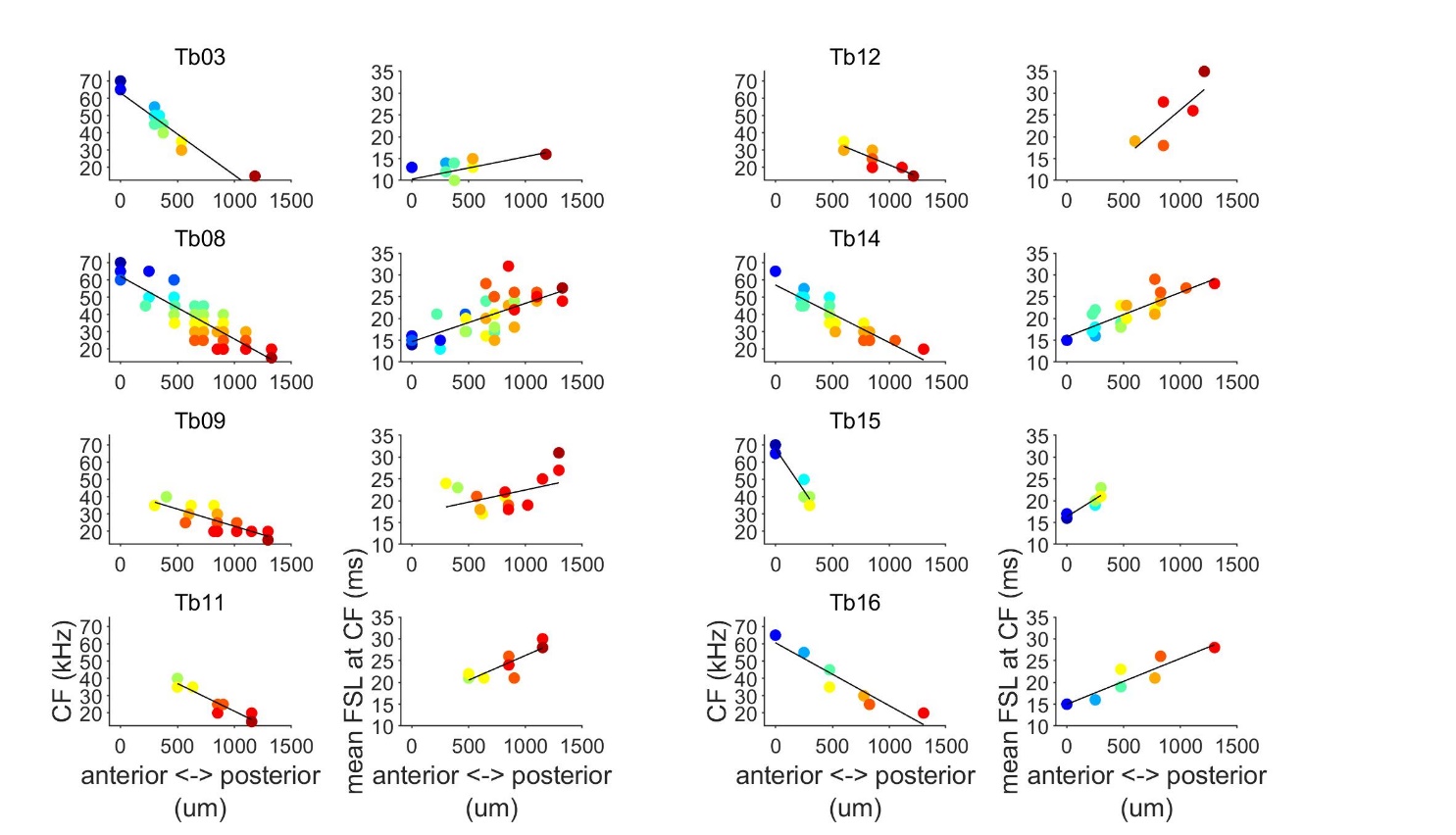


**S1. Fig. Topography of characteristic frequency (CF) and mean first spike latency (FSL) in the A1.** CF and mean FSL as a function of the cortical anterior-posterior position in each bat included in this study. CF is color coded similar to Fig. 2. Black lines represent the best fitted curves of the linear regression analyses. Data underlying this figure can be found at https://doi.org/10.18738/T8/GLVN1J
