# Supplementary material for: Temporal coding of echo spectral shape in the bat auditory cortex
Source: PLoS Biol. 2020 Nov 10;18(11):e3000831. doi: 10.1371/journal.pbio.3000831 (PMC7678962; doi:10.1371/journal.pbio.3000831)
Supplement: S2 Fig — Activation pattern profile and mean FSL in each bat in response to flat-spectrum (left), 30-kHz notched (center), and 45-kHz notched (right) dFM of each bat. Neurons are tonotopically organized. Mean FSL was calculated from the response of neurons with equal CFs. CF is color coded following Fig 2. Data underlying this figure can be found at https://doi.org/10.18738/T8/GLVN1J. A1, primary auditory cortex; CF, characteristic frequency; dFM, downward frequency-modulated sweep; FSL, first-spike latency. (DOCX) [file pbio.3000831.s002.docx]

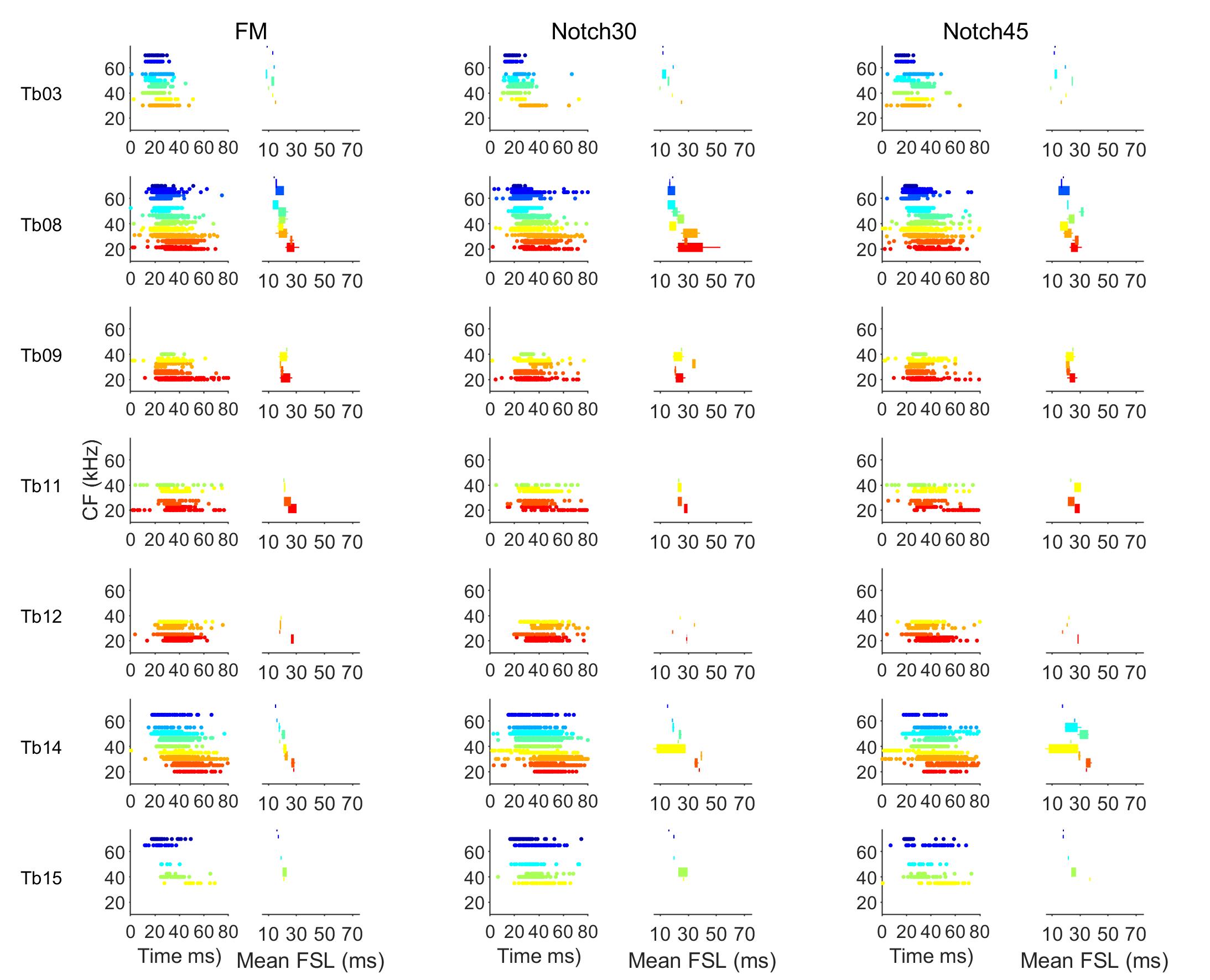


**S2. Fig. Sequential activation of the A1.** Activation pattern profile and mean FSL in each bat in response to flat-spectrum (left), 30 kHz notched (center) and 45 kHz notched (right) dFM of each bat. Neurons are tonotopically organized. Mean FSL was calculated from the response of neurons with equal CFs. CF is color coded following Fig. 2. Data underlying this figure can be found at https://doi.org/10.18738/T8/GLVN1J
