# Supplementary material for: Temporal coding of echo spectral shape in the bat auditory cortex
Source: PLoS Biol. 2020 Nov 10;18(11):e3000831. doi: 10.1371/journal.pbio.3000831 (PMC7678962; doi:10.1371/journal.pbio.3000831)
Supplement: S3 Fig — Matrices were calculated in response to flat-spectrum (left), 30-kHz notched (center), and 45-kHz notched (right) dFM of each bat. Synchronization index (c) range from 0 (blue, no spike synchrony) to 1 (red, maximum spike synchrony). Data underlying this figure can be found at https://doi.org/10.18738/T8/GLVN1J. dFM, downward frequency-modulated sweep. (DOCX) [file pbio.3000831.s003.docx]

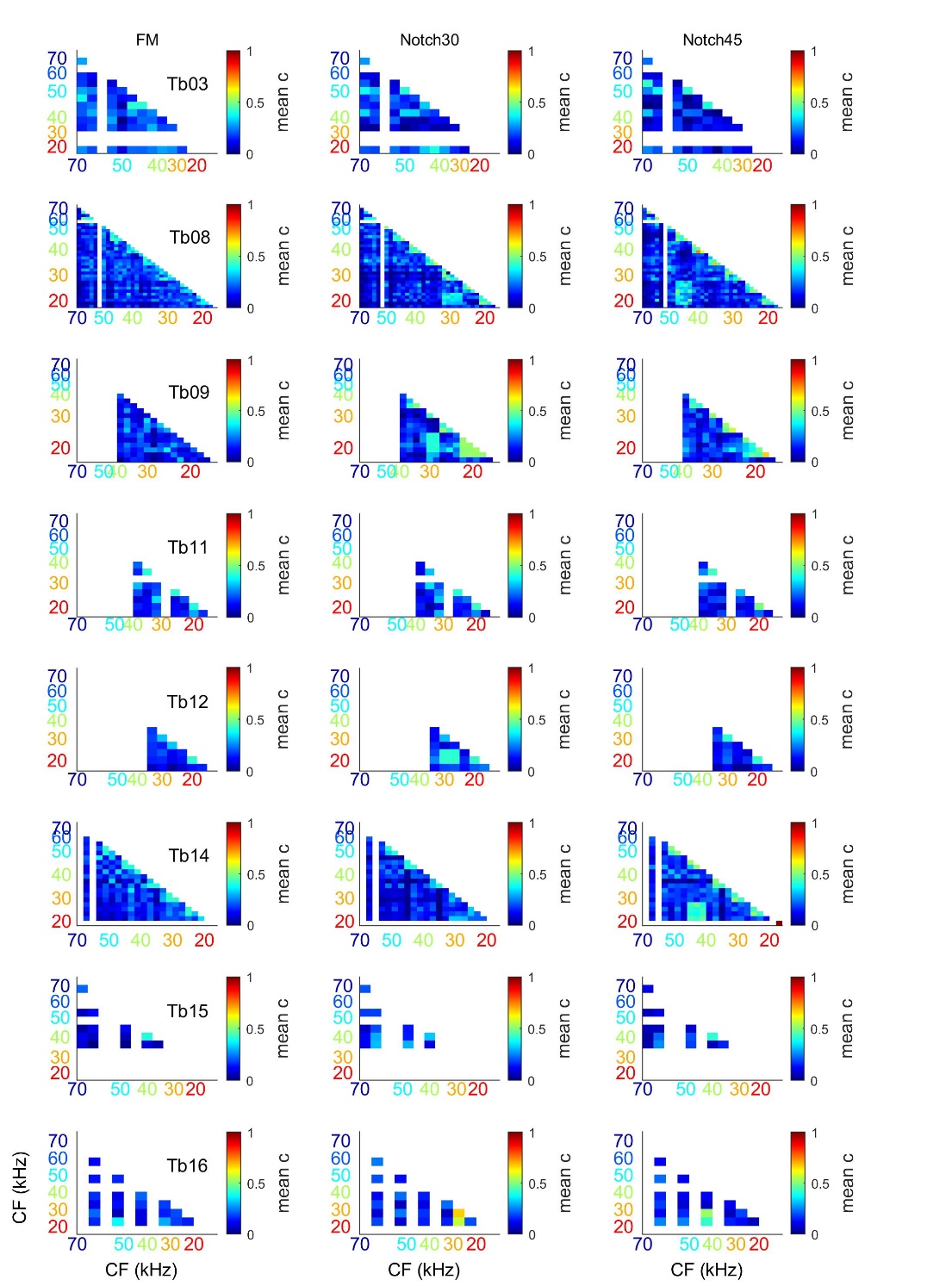


**S3. Fig. Synchronization matrices.**  Matrices were calculated in response to flat-spectrum (left), 30 kHz notched (center) and 45 kHz notched (right) dFM of each bat. Synchronization index (c) range from 0 (blue, no spike synchrony) to 1 (red, maximum spike synchrony). Data underlying this figure can be found at https://doi.org/10.18738/T8/GLVN1J
